# miRFANs: an integrated database for *Arabidopsis thaliana* microRNA function annotations

**DOI:** 10.1186/1471-2229-12-68

**Published:** 2012-05-14

**Authors:** Hui Liu, Ting Jin, Ruiqi Liao, Linxia Wan, Bin Xu, Shuigeng Zhou, Jihong Guan

**Affiliations:** 1 Research Lab of Information Management, Changzhou University, Jiangsu, China; 2 School of Computer Science, Fudan university, Shanghai, China; 3 Department of Computer Science & Technology, Tongji University, Shanghai, China; 4 Shanghai Key Lab of Intelligent Information Processing, Fudan University, Shanghai; 5 School of Information Science and Technology, Hainan University, Hainan, China

## Abstract

**Background:**

Plant microRNAs (miRNAs) have been revealed to play important roles in developmental control, hormone secretion, cell differentiation and proliferation, and response to environmental stresses. However, our knowledge about the regulatory mechanisms and functions of miRNAs remains very limited. The main difficulties lie in two aspects. On one hand, the number of experimentally validated miRNA targets is very limited and the predicted targets often include many false positives, which constrains us to reveal the functions of miRNAs. On the other hand, the regulation of miRNAs is known to be spatio-temporally specific, which increases the difficulty for us to understand the regulatory mechanisms of miRNAs.

**Description:**

In this paper we present miRFANs, an online database for *Arabidopsis thaliana*miRNA function annotations. We integrated various type of datasets, including miRNA-target interactions, transcription factor (TF) and their targets, expression profiles, genomic annotations and pathways, into a comprehensive database, and developed various statistical and mining tools, together with a user-friendly web interface. For each miRNA target predicted by psRNATarget, TargetAlign and UEA target-finder, or recorded in TarBase and miRTarBase, the effect of its up-regulated or down-regulated miRNA on the expression level of the target gene is evaluated by carrying out differential expression analysis of both miRNA and targets expression profiles acquired under the same (or similar) experimental condition and in the same tissue. Moreover, each miRNA target is associated with gene ontology and pathway terms, together with the target site information and regulating miRNAs predicted by different computational methods. These associated terms may provide valuable insight for the functions of each miRNA.

**Conclusion:**

First, a comprehensive collection of miRNA targets for *Arabidopsis thaliana* provides valuable information about the functions of plant miRNAs. Second, a highly informative miRNA-mediated genetic regulatory network is extracted from our integrative database. Third, a set of statistical and mining tools is equipped for analyzing and mining the database. And fourth, a user-friendly web interface is developed to facilitate the browsing and analysis of the collected data.

## Background

miRNAs are a class of short (∼22 nucleotide) noncoding RNAs expressed in both animal and plant cells. These small RNA molecules bind preferably to the 3’ untranslated region (3’UTR) of protein coding genes and make them degradation and/or translation inhibition [1]. A typical miRNA target site has perfect or nearly perfect complementarity to the miRNA so-called seed sequence, i.e., ∼7 nucleotides from miRNA 5’ end [2,3]. Both experimental and computational studies have shown that miRNAs are a class of post-transcriptional regulators, and play important roles in cellular process [4,5], developmental control [6,7], hormone secretion [8], cell differentiation and proliferation [9], and responses to environmental stresses [10,11]. One challenge facing miRNA research is to accurately identify the target mRNAs, because of the very limited sequence complementarity between miRNAs and their targets, and the scarcity of experimentally validated gene targets to guide accurate prediction models [12].

*Arabidopsis thaliana*, a small flowering plant with only several weeks of life cycle, has important advantages for the research of genetics and molecular biology, thus some special databases for *Arabidopsis thaliana* have been established. TAIR is a comprehensive information system that deposits genome, expression profiling, proteome, pathway and functional annotations, together with many analysis and visualization tools [13]. For miRNAs, Sanger Institute’s miRBase serves as the central depository where miRNAs are experimentally validated. The current release, version 10.0, contains over 266 *Arabidopsis thaliana* miRNAs [14]. However, the functions of most miRNAs are still unknown so far. The Arabidopsis Small RNA Project Database, abbreviated as ASRP, intends for *Arabidopsis thaliana* small RNA mature sequences, transcripts and locus [15]. PMRD (Plant miRNA Database) has collected miRNA sequences, miRNA target genes and expression profiles of some model plants [16]. mirEX [17] collects information about the expression profiles of 190 *Arabidopsis thaliana* pri-miRNAs in seven different developmental stages and provides simultaneous comparison of expression levels between various microRNA genes in diverse organs and developmental stages.

However, all databases mentioned above pay main attention to collecting miRNA-related information such as miRNA sequences, miRNA-target bindings. No system has been devoted to the functional exploration of miRNAs. Even though a few databases include expression information of miRNAs, the coverage is quite limited, failing to integrate most of the microarray and high-throughput experimental results. The recently published database miRNEST [18] is a comprehensive collection of animal, plant and virus microRNA-related data, including miRNA sequence, expression, polymorphisms and targets data, but it has no function annotations and pathways data, and does not provide comparative analysis of expression profiles from identical tissues or samples between miRNAs and their targets.

In this paper, we introduce miRFANs, an integrated database with a friendly web interface for functional annotations of miRNAs, which encompasses miRNA-target interactions, expression, functional annotations, pathway terms. For miRNA targets, three predicted sets by three computational algorithms (psRNATarget [19], TargetAlign [20] and UEA target-finder [21]) and two experimentally validated sets (miRTarBase [22] and TarBase [23]) are collected. However, the miRNA-target interactions predicted by computational methods only imply the possibility that miRNAs regulate their targets under certain spatio-temporal conditions, as the regulation of miRNAs is well known to be condition- and tissue-specific. The expression data provides us the chance to evaluate the effect of miRNA binding in the sense of a certain environmental condition and tissue, on the basis of several evidences showing that the expression profiles of miRNAs are closely related to that of their target genes. The reciprocal expression patterns between miRNAs and their targets can be remarkable evidence of miRNA regulatory events. Therefore, we download expression datasets of both miRNAs and genome from the gene expression omnibus (GEO) [24]. Expression datasets are preprocessed and integrated for readily identifying co-expressed or differentially expressed mRNAs and their targets. We also developed a web interface supporting diverse query entries that include search by miRNA, expression profile or pathway, and a mining toolbox including correlation, differential expression analysis and clustering to explore the expression data.

Thus, miRFANs can serve as a comprehensive resource for exploring the functions of *Arabidopsis thaliana* miRNAs.

## Construction and content

### Data sources and preprocessing

In what follows, we introduce the data sources and preprocessing procedures for constructing the database.

#### miRNAs and miRNA targets

Mature miRNA sequences are downloaded from miRBase database (release 17), which includes 266 miRNAs of *Arabidopsis thaliana*, the whole genome sequences are obtained from TAIR.

miRNA target genes are acquired by four ways: 1) experimentally validated miRNA target genes from two manually curated databases, TarBase [23] and miRTarBase [22]. For convenience, we combine them into one dataset denoted by (miR)TarBase that includes 81 miRNA-target interactions; 2) miRNA targets predicted by psRNATarget [19], which uses the scoring schema originally applied by miRU [25] to evaluate the complementarity between small RNA and target gene transcript, and utilize RNAup included in Vienna Package [26] to estimate the target site accessibility; 3) miRNA targets predicted by TargetAlign [20], a Smith-Waterman-like alignment tool that uses dynamic programming to build a score matrix based on the complementarity of nucleotides for tracing the optimal local alignments; and 4) miRNA targets predicted by target-finder developed by UEA lab [21], which tries to predict miRNA target genes from high-throughout sequencing data. To the best of our knowledge, miRFANs is the most comprehensive miRNA targets database up to date. For more details of the data please see Additional File 1.

#### Expression profiles

The miRNA expression profiles are obtained from high-throughout sequencing datasets downloaded from Gene Expression Omnibus (GEO), ASRP and PMRD. These datasets are normalized by using miRAnalyzer [27], which adopts a fast, short read alignment algorithm Bowtie [28] to map the short reads to mature miRNA sequences, and counts the number of mapped reads for each miRNA. miRAnalyzer outputs both the read count and normalized value for each miRNA. The normalized value is computed as read count which is first divided by the read count of all unique reads mapped to this library and then times 100. In total, the miRNA expression datasets include 12 experiments, 81 samples and 13 types of tissues.

The genome-wide expression profiles are downloaded from GEO [24], the datasets include 548 microarray experiments, 6740 samples, 53 types of tissues. For single channel data, expression levels are normalized to count data, reflecting the relative measure of abundance of each transcript. For dual channel experiments, expression levels are normalized to log ratios. For low quality or absent signals, we run KNNImpute [29] to estimate the expression levels.

#### TFs and target genes

As a gene may be regulated by both TFs and miRNAs, integration of the regulation relationship between TFs and their target genes into the database will give more insight into the combinatorial regulation of miRNAs and TFs to their common target genes. We download the TF and regulatory network datasets from AGRIS [30,31] website and integrate them into our database.

#### GO and pathways

To explore the functional annotations and pathways of miRNA targets, we download the whole genome annotations from GO [32] and pathways from KEGG [33].

**Figure 1:**
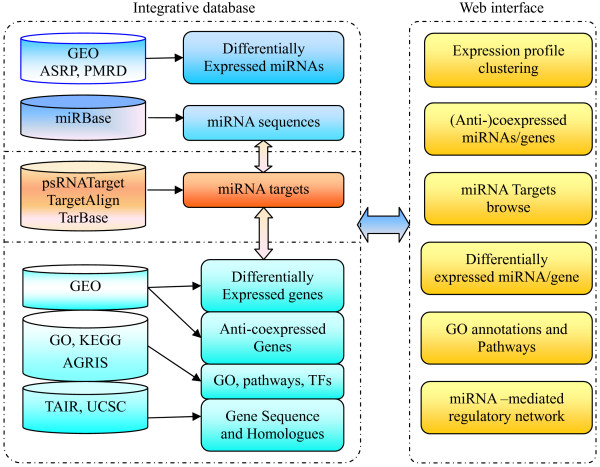
**Data sources and architecture of miRFANs.** The architecture of miRFANs. Left: data sources and components of the database; Right: datasets accessible from the web interface.

We integrate all the data mentioned above by designing a new database schema, and thus obtain a comprehensive database for *Arabidopsis thaliana* miRNAs and their targets. The flowchart of the data source and integration process is shown in Figure 1.

### Comparison of differential expression

In general, the interactions between a miRNA and its targets predicted by computational methods imply merely the possibility that the miRNA regulates the targets under a certain spatio-temporal condition, as the regulation of miRNA is well known to be condition- and tissue-specific. However, up to now we still know little about in which tissues, to what environmental stimuli and on which development stages miRNAs regulate their targets.

**Figure 2:**
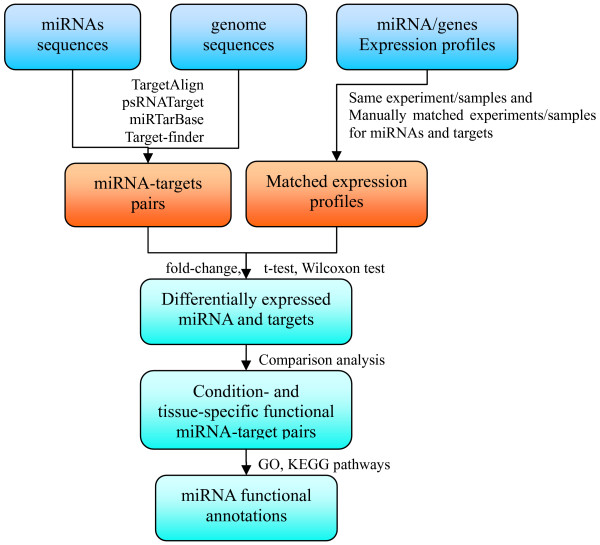
**The workflow of differential expression comparison.** The workflow of differential expression comparison between miRNAs and their targets, based on the samples of similar (or manually matched) environmental conditions and tissues.

The major goal of miRFANs is to tackle the problem by conducting differential expression comparison between miRNAs and their targets under the same (or similar) experimental condition and in the same tissue, so that we can identify the specific spatio-temporal conditions under which the true regulation events happen. Specifically, we choose the experiments that measure expression profiles for both miRNAs and genes from the same samples, and also manually matched the expression profiles between miRNAs and their targets according to the experimental platform, plant growth and treatment condition and tissue. We then do differential expression comparison between miRNAs and their targets based on each pair of samples to check whether or not significantly up-regulated (or down-regulated) miRNAs lead to down-regulation (or up-regulation) of the targets. This is helpful for us to reveal the tissues, environmental conditions in/under which miRNAs do significantly repress their targets, so as to deepen our understanding of the mechanism of miRNA regulation. Figure 2 illustrates the workflow of differential expression comparison.

### miRNA-mediated pathways

In order to investigate the functional roles played by miRNAs, we integrate them into metabolic pathways to identify their effect on the downstream genes. As DIANA-mirPath [34], the enrichment analysis of each pathway mediated by a miRNA is performed by Pearson’s Chi-squared test ( $\chi^{2}=\sum ({\left(O-E\right)}^{2}/E)$), where *O* (Observed) is the number of genes in the input dataset found to participate in a given pathway, and *E* (Expected) is the number of genes expected by chance to be member of that pathway, given the pathway and input list size. The KEGG pathways involved by a miRNA of interest is ranked by the negative natural logarithm of the *p*-value ( −lnp).

### miRNA-mediated genetic regulatory network

Based on the miRNA-target and TF-target interactions, we extract a miRNA-mediated regulatory network according to the following rules: 1) all miRNA-target interactions recorded in TarBase and miRTarBase are included. 2) only TFs with more than 10 GO terms are take into account so as to obtain an informative regulator network. 3) As miRNAs generated from the same miRNA family usually regulate common targets, miRNAs are represented by miRNA families so that the regulatory network is compact and can be well demonstrated via web-interface. As a result, we get a miRNA-mediated genetic regulatory network with 12 miRNA families, 41 TFs and 155 regulation relationship among them.

## Utility and Discussion

### Web interface

A user-friendly web interface is implemented to browse and analyze these data. Specifically, we develop two display modes, interactive table and summary text, to facilitate the data presentation and mining operators. In summary text mode, miRFANs shows summary information of a miRNA or a gene of interest in plain text, including the sequence, target genes, samples in which the miRNA or protein-coding gene is up-regulated and down-regulated. Moreover, the TF family information, GO and pathway are also be shown for any protein-coding gene. In interactive table mode, all data are organized into tables. Each row of the table shows the number of target genes and samples with respect to a miRNA and a gene. Detail will be dynamically displayed when user click these numbers. Note that only in interactive table mode the analysis toolbox can be launched to carry out profiling, clustering, differential expressed analysis etc.

The database is developed using Microsoft SQL Server 2005 and consists of 19 physical tables (see Additional File 2: Description of the physical data model of the miRFANs database). The architecture of the server is designed by using Spring2.5+Struts2.0. The Web interface is developed by using XHTML, JSP, JavaScript, Jquery, Ajax and CSS. Both CSS and XHTML scripts respect the W3C standards and are validated by W3C online tools. Moreover, several visualization tools are employed to show the expression profiles, clustering, differential expressed comparison and miRNA-mediated genetic regulatory network. JHeatChart^a^, a Java library for generating heat map charts is used to show the result of differential expressed analysis. Highcharts^b^ is a JavaScript charting library and is suitable for illustrating the expression profiles. we also exploit Java TreeView^c^ to show the result of clustering and Cytoscape Web^d^ to show miRNA-mediated genetic regulatory network.

### Search

We develop a search module to support query for a miRNA or a coding gene of interest. For miRNAs, each of the two display modes mentioned above can be used to show the search result, and miRNA name (such as ath-miR156a) or miRNA family name (such as ath-miR156 or miR156) can be taken as search keyword. When miRNA family name is input, the first matched miRNA of that miRNA family is used to guide the subsequent search process, and other miRNA members will also be listed as hyperlinks. For coding gene, only summary text display mode can be used at present and we will proceed to develop interactive table mode. For any miRNA, gene, GO term or metabolic pathway presented in the search result page, we set hypelinks to various well-known biology databases such as miRBase, TAIR, NCBI, KEGG.

### Analysis toolbox

To facilitate the expression profiles analysis, we have developed an analysis toolbox that contains a number of classical expression profiles analysis and visualization functions. These functions include 1) identifying co-expressed (or anti-coexpressed) miRNAs/genes through computing Pearson correlation coefficients, Spearman correlation coefficients, cosine similarities and KL divergences based on the expression profiles of interest; 2) discovering differentially expressed miRNAs/genes via fold-change, t-test, Wilcoxon test, etc. 3) clustering analysis such as k-means, hierarchical clustering.

**Figure 3:**
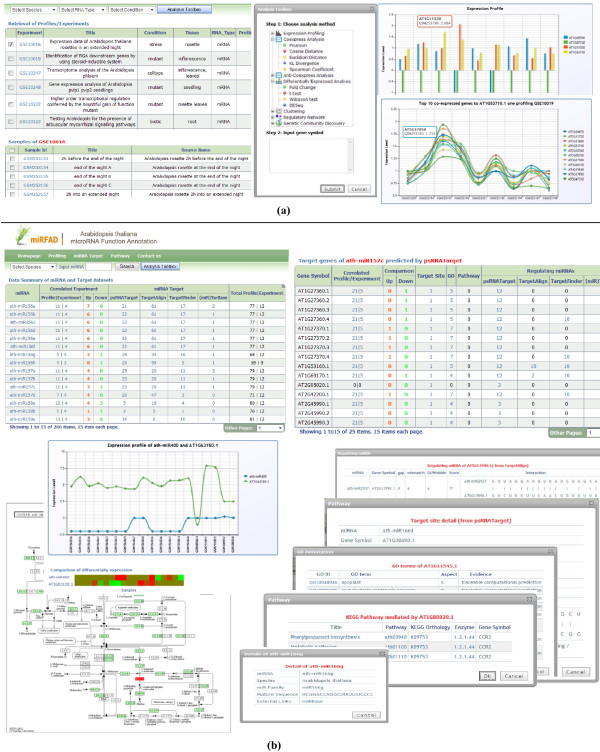
**The screenshots of miRFANs.** The screenshots of miRFANs. **(a)** Retrieval and statistical analysis of expression profiles for both miRNAs and target genes. Several types of expression analysis, including (anti-)coexpression, clustering, differential expression analysis, can be conducted by the toolkits. **(b)** Functional annotations of miRNAs by integrating the differential expression comparison and the GO as well as pathway terms.

Figure 3(a) shows screenshots of the profiling module which is developed to browse and analyze the expression profiles by launching the Analysis toolbox. Figure 3(b) shows the screenshots of the functional annotation and pathway modules.

### Future work

Further development of miRFANs will be mainly focused on at least three aspects. First, we plan to integrate miRNAs into transcriptional regulatory networks and thus construct miRNA-mediated regulatory networks for *Arabidopsis thaliana*. Second, we will include more analysis functions, such as building regulatory networks and genetic community discovery, into the analysis toolbox. Finally, we will continuously collect data and integrate other model plants into miRFANs.

## Conclusion

miRFANs is an integrative database of *Arabidopsis thaliana* miRNAs and their target genes, expression profiles, function annotations and pathways. A friendly web interface is developed to browse and analyze of the data. We believe that miRFANs is a useful platform for exploring the regulatory functions of *Arabidopsis thaliana* miRNAs and can provide considerable value for many researchers.

## Availability and requirements

miRFANs is freely available at http://www.cassava-genome.cn/mirfans. In the development of miRFANs, we follow the ordinary standards of web applications, and the Java scripts employed are cross-browser scripts. We have confirmed that miRFANs can work well with three web-browsers, Google Chrome, Microsoft Internet Explorer and Mozilla Firefox, with no need for any plugins or special system requirements.

## Endnotes

^a^http://www.javaheatmap.com/

^b^http://www.highcharts.com/

^c^http://jtreeview.sourceforge.net/

^d^http://cytoscapeweb.cytoscape.org/

## Author’s contributions

HL designed the server and web interface, developed the profiling and miRNA target modules, and wrote the manuscript. TJ collected the GO and pathway data, developed the pathway module. LW and RL collected and preprocessed the expression data, and participated in the design of the relational database scheme. BX implemented the development of toolbox, and is also responsible for the update and maintenance of the database. SZ and JG provided support and participated discussions on the design and development of the database, coordinated the whole project and revised the manuscript.

## Supplementary Material

Additional file 1 **Sample additional file title.** Detail of the datasets integrated into miRFANs database. Click here for file

Additional file 2 **Description of the relational database schema of miRFANs.** The database is built on Microsoft SQL Server 2005 and includes 19 physical tables. The relational database schema is automatically generated using ModelRight 3.5 professional through reverse engineer from the database. Click here for file
