# Supplementary material for: miRFANs: an integrated database for Arabidopsis thaliana microRNA function annotations
Source: BMC Plant Biol. 2012 May 14;12:68. doi: 10.1186/1471-2229-12-68 (PMC3489716; doi:10.1186/1471-2229-12-68)
Supplement: Additional file 1 — Sample additional file title. Detail of the datasets integrated into miRFANs database. [file 1471-2229-12-68-S1.doc]

**Additional File 1**

Mature miRNA sequences are downloaded from miRBase database (release 17), the whole genome sequences are obtained from TAIR. miRNA target genes are acquired from two experimentally validated miRNA target genes databases, TarBase and miRTarBase, and predicted miRNA targets by psRNATarget, TargetAlign, Target-finder. Both microarray and high-throughput sequence datasets are downloaded from GEO, ASRP, TAIR. GO annotations and pathways are download from Gene Ontology and KEGG, respectively. The detail of the datasets are shown in the table 1.

Table 1 Details of datasets

| miRNA mature sequence | | 266 | |
| --- | --- | --- | --- |
| Expression Profile | | 12 experiments, 81 samples covering 13 types of tissues | |
| Gene sequence | | 28049 | |
| Expression Profile | | 548 experiments, 6740 samples covering 53 types of tissues | |
| TFs and Target genes | | 1851 TFs and 11355 TF-target interactions | |
| Target gene | | (miR)TarBase | 81 interactions |
|  |  | psRNATarget | 251 miRNAs, 1546 targets and 2415 interactions |
|  |  | Target-align | 237 miRNAs, 3297 targets and 5159 interactions |
|  |  | Target-finder | 224 miRNAs, 1135 targets and 1778 interactions |
| GO terms | 190994 GO annotations, 4528 unique GO terms | | |
| KEGG pathway | 14786 gene-pathway records, 124 unique pathways | | |
