# Supplementary material for: miRFANs: an integrated database for Arabidopsis thaliana microRNA function annotations
Source: BMC Plant Biol. 2012 May 14;12:68. doi: 10.1186/1471-2229-12-68 (PMC3489716; doi:10.1186/1471-2229-12-68)
Supplement: Additional file 2 — Description of the relational database schema of miRFANs. The database is built on Microsoft SQL Server 2005 and includes 19 physical tables. The relational database schema is automatically generated using ModelRight 3.5 professional through reverse engineer from the database. [file 1471-2229-12-68-S2.doc]

**Additional File 2**

To integrate all datasets acquired from different databases, we designed a flexible relational database scheme, as shown in the figure 1. We use the miRNA name as the primary key of the tables related to miRNA sequence, expression profile, and targets, and use Arabidopsis Genome Initiative identifier (AGI_ID) as the primary key of the tables related to gene sequence, expression, GO and pathway terms. The database scheme contains 19 physical tables. We also build a view to transfer the expression profiles contained in table [expression] into a matrix form for each experiment, i.e., each row is a miRNA or gene and each column is a sample of the experiment. To tune the performance of the database we materialized all these views. Fig.1 is automotive generated using ModelRight 3.5 professional through reverse engineer from Microsoft SQL Server 2005 database that stores our data.


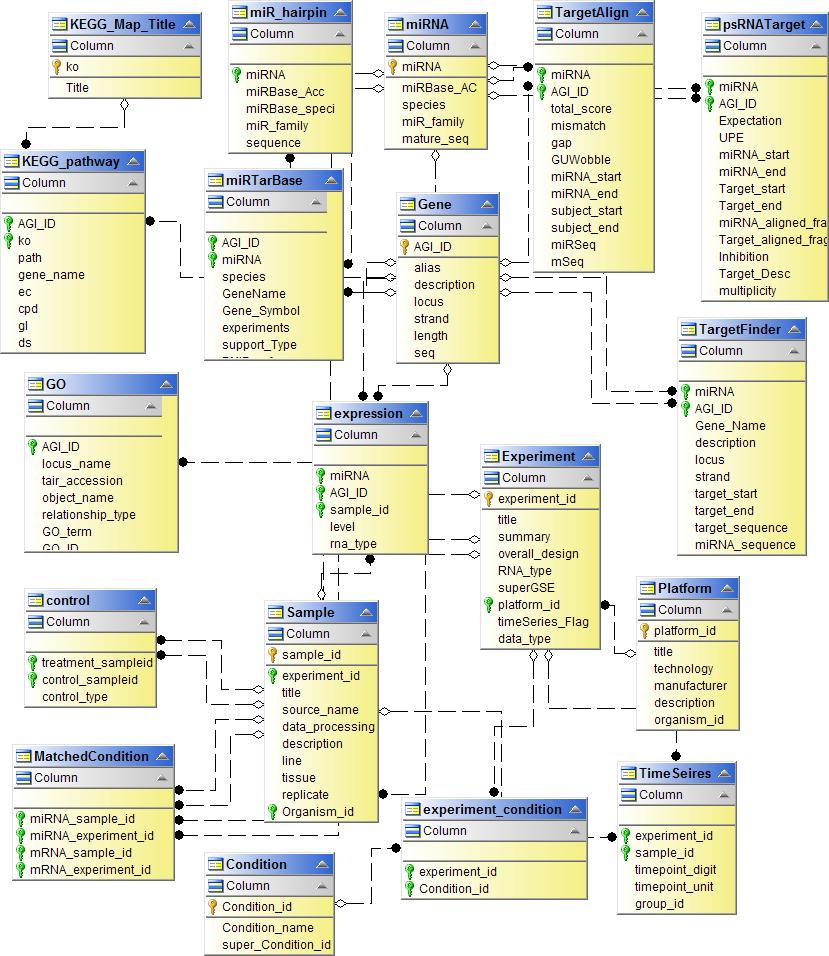


Fig.1 Relational database scheme of miRFAD.
